# 

**Published:** 1862-06

**Authors:** 


### CHICAGO MEDICAL JOURNAL.

**Terms, $2.00 per Annum.**

### CONTENTS OF THE JUNE NUMBER.

SELECTED.

PAGE.
On the Temperature, Urea, Chloride of Sodium, and Urinary Water in Scarlet Fever, and on a Cycle in Disease and Health. By Sydney Ringer, M. D .......... 315
Influence of Railway Traveling upon Public Health .......... 318
Action of the Second Class of Voluntary Muscles .......... 326
General Order in Reference to Sanitary Precautions .......... 335

ORIGINAL COMMUNICATIONS.

Cases in Ophthalmic Practice. By E. L. Holmes, M. D., of Chicago..... 338
A Case of Eclampsia .......... 341
EDITORAL AND MISCELLANEOUS .......... 346

